# The Reform That Was Never Completed: Why Greece Must Redesign Its Health Financing Architecture

**DOI:** 10.3390/healthcare13243213

**Published:** 2025-12-08

**Authors:** Angeliki Flokou, Vassilis Aletras, Dimitris A. Niakas

**Affiliations:** 1 Medical School, National and Kapodistrian University of Athens, 11527 Athens, Greece; dimitris.niakas@gmail.com; 2 School of Social Sciences, Hellenic Open University, 5 Evdoxou Str., 26331 Patra, Greece; 3 Department of Business Administration, School of Business Administration, University of Macedonia, 156 Egnatia Str., 54636 Thessaloniki, Greece; valetras@uom.edu.gr

**Keywords:** health financing, Greece, universal health coverage, out-of-pocket payments, financial protection, equity in access, unmet health care needs, single purchaser model

## Abstract

Health financing is a core determinant of the resilience and equity of health systems. Using WHO’s three-pillar framework as an orienting reference—rather than a prescriptive template—this article analyzes the evolution, structural shortcomings, and policy dilemmas of the Greek health financing model, within a comparative European context. While many EU countries have strengthened public financing to ensure universal access, Greece maintains a hybrid, fragmented model in which out-of-pocket payments play a disproportionately large role. Despite recurrent reform attempts, Greece has not developed a cohesive public system with a clear commitment to social solidarity. Instead, the system has silently shifted into a de facto semi-privatized two-tier model that exacerbates social inequities, limits access and undermines efficiency. Drawing on international experience and documented policy lessons, the article proposes a strategic redesign of the health financing architecture. The proposal is conceptual and does not enter implementation specifics. Its central axis is the establishment of two national single purchasers of health services by level of care, with a clear allocation of responsibilities and authority, the Ministry of Health for hospital care, and the National Organization for Healthcare Services Provision (EOPYY) for primary, outpatient, and post-acute/rehabilitation care, to strengthen prevention, equitable access, and chronic care management while easing pressure on hospitals. The proposed model includes targeted investments in human resources and infrastructure, the enhancement of prospective payment mechanisms, the strengthening of primary care networks, and the leveraging of innovation. At the same time, it provides for reforms in governance, digital transformation of the system, and reallocation of resources based on principles of equity and efficiency. The proposed overall restructuring aims to strengthen financial protection, reduce inequities in access, and improve health outcomes through a publicly oriented, socially responsive, and strategically governed system.

## 1. Introduction

When needs exceed available resources, the health care system becomes a mirror of a society’s values, revealing whom it prioritizes and whom it leaves behind. That choice depends not only on the level of funding, but also on political choices, institutional arrangements, and the extent to which health care is treated as a public good. Here, health systems must balance equity, efficiency, and the financial protection of the population. Given limited resources, all countries—whatever their income level—should focus on making the most of them to achieve their stated goals and to maintain fiscal sustainability in the context of universal health coverage [1]. According to the World Health Report, the general objectives of health systems are to improve health and health equity, respond to the needs of the population with financial fairness, and make effective use of available resources [2]. Alongside the general objectives, there are also intermediate, no less necessary, objectives, such as ensuring equal access for equal needs, enhancing transparency, improving quality, and making services more efficient in the provision of health services and in system administration [3]. These objectives are interrelated and, to a large extent, complementary. For example, although achieving equitable access can require extra resources, the gains from increased efficiency can offset them, provided they are reinvested in the system [3,4,5].

Despite facing common challenges, national health systems differ, reflecting country-specific needs, institutions, and histories [6]. All systems combine public and private roles in financing and provision; configurations vary by country, but none is purely public or purely private [7]. Social, demographic, economic and environmental conditions both shape population health and are, in turn, shaped by the health system’s organization and performance [8]. In this context, structural factors (system design, infrastructure, workforce), political–institutional arrangements (governance, regulation, priority-setting), and behavioral dynamics (care-seeking, patient expectations, provider incentives) interact with the financing functions to condition access, equity, and efficiency, with implications for sustainability and resilience [8,9,10,11]. These determinants ultimately shape not only financial indicators, but also the value delivered to patients in terms of access, financial protection and health outcomes. Against this background, Greece represents a salient case of a hybrid financing model with persistently high out-of-pocket spending, unmet needs, and fragmented purchasing arrangements.

This viewpoint paper aims to analyze the current state of health financing in Greece using official data and relevant indicators to identify systemic weaknesses, such as disproportionate private spending, high levels of unmet medical needs, and ongoing health disparities. These problems reflect the incomplete results of past reforms and highlight the need for a fundamental overhaul of the country’s health financing system. As a distinctive contribution, the paper advances a focused reform direction—two national single purchasers by level of care (the Ministry of Health for hospital care; the National Organization for Healthcare Services Provision, EOPYY, for primary and post-acute care)—used as an organizing lens for the subsequent analysis. The paper also presents key principles and directions for this redesign, striving to align equity, access, and sustainability within a more cohesive system.

The paper is organized as follows: Section 2 (Determinants of health systems and financing functions) outlines structural, political–institutional, and behavioral drivers, and briefly situates demand (needs) vs. supply (capacity) and their interaction with financing in shaping access, equity, and efficiency. Section 3 (Characteristics of the Greek Health System) describes the system’s main features; Section 4 (Health Expenditure Indicators) reviews spending metrics. Section 5 (the price of inaction: high private spending, low protection) examines consequences, with Section 5.1, Section 5.2, Section 5.3, Section 5.4 and Section 5.5 on unmet needs/inequalities, dental care, informal payments and medicines, PHC challenges and the hospital-centered model, and trust/readiness/digital maturity. Section 6 (Conclusions and recommendations) summarizes key messages and policy recommendations to support the comprehensive and sustainable reform of Greece’s health financing system.

## 2. Determinants of Health Systems and Financing Functions

A health care system can be viewed as a “two-sided coin”, as it includes two distinct but interdependent dimensions: on the one hand, the demand for and financing of health services and, on the other, their supply and provision. Both sides are inextricably linked and the system works well only when they stay in balance and meet the needs of the population [12,13]—a balance that should not be left to market mechanisms alone, as the health sector is governed by particularities that render the logic of competition and any automatic balancing of supply and demand inadequate. A critical regulator is the reimbursement mechanism, which shapes the flow of funding and the provision of services in the system. Funding mechanisms are commonly divided into interventions that, on the demand side, aim to lower financial barriers to access and encourage the use of services (e.g., zero copayment for preventive examinations), while on the supply side, they set incentives to improve access and quality of services for target populations [14]. In this context, financing is a key factor for the proper functioning of the health system, as documented in the international literature [15]. An effective financing model secures sufficient resources, ensures access to necessary services, protects individuals from excessive costs and catastrophic expenses, and aligns the incentives of providers and users for efficient use [2,16,17]. In health systems where third-party payers play a major role, this can create scope for adverse behaviors, such as demand-side moral hazard (consumer behavior) and/or supplier-induced demand (prescriber behavior), which further distort the system and undermine both efficiency and equity.

Therefore, financing is not merely a mechanism for transferring resources but is central to achieving the fundamental goals of any health system, such as Universal Health Coverage (UHC), one of the United Nations Sustainable Development Goals [18]. UHC requires balanced progress across three dimensions—breadth (who is covered), scope (which services are covered), and depth (what share of costs is covered from pooled/public funds)—see (Table 1) [19,20]. These dimensions are closely tied to health financing policy and to the monitoring of progress toward UHC [3]. To move closer to universal coverage, countries need to cover more people, include more services, and/or strengthen financial protection by reducing out-of-pocket payments [21]. The balance between these dimensions is not just technical but also a deeply social—and political—as it reveals whom the system cares for and whom it leaves out.

Countries adopt different financing schemes, tailored to their economic, social, and political context, prioritizing universal coverage and protection of vulnerable population groups [15,22]. That said, the effectiveness of these options hinges on the level of socioeconomic development and on intrasystemic characteristics that shape how well the system functions and its quality [21]. Historical development, political context, and physical geography shape the organization and functioning of health systems. Greece, due to its mountainous mainland and numerous islands, has many geographically isolated areas that often lack specialized staff and facilities, compelling residents to travel for care or rely on private practitioners [23,24].

The political dimension is vital, shaping resource collection and distribution, the health care market, the creation of service delivery guidelines, and, more broadly, strategy development to ensure access and quality. In the “former socialist countries of Europe” (FSE), the primary method of financing depended largely on tax-funded state revenues under a budgetary Semashko state model [25,26,27]. After the socialist regime fell, most countries carried out extensive reforms, transitioning to a Bismarck-style compulsory social insurance system [25,26].

In Russia, for example, the Mandatory Health Insurance (MHI), introduced in 1991, sought to raise funding, foster provider competition, strengthen patient protection, and improve resource use, financing it primarily through mandatory employer and regional contributions rather than the state budget [26]. While it enhanced funding sustainability and a fairer allocation of resources, significant shortcomings persist in care quality, efficiency gains, and the development of new medical technologies [26,27,28]. More broadly, European health systems are often categorized as Beveridge-type, Bismarck-type, and mixed or hybrid models, reflecting different historical paths and political decisions in health financing.

Most analyses focus on structural and political factors, but behavioral factors, though important, are often overlooked. Patients’ choices reflect a complex interplay between their own characteristics and those of health care providers [29]. Even when people spend significant time searching for information, many still struggle to compare available options and make fully informed decisions [29]. Their behavior is shaped not only by objective factors but also by established habits and social perceptions, which vary across different population groups and influence how health services are judged and accepted [30]. Furthermore, while guidelines for achieving universal health coverage often emphasize capacity expansion and financing options, they may underestimate the socio-cultural context necessary for the success of such strategies. Understanding the local socio-cultural environment—including gender norms and social roles—is crucial for explaining access patterns [9]. For example, in Greece and other Mediterranean countries, traditional family care is deeply embedded in the social fabric and often compensates for gaps in formal health and social care. This strong family model (symbiosis, intergenerational solidarity, predominantly female caregivers), reinforced by economic pressures, makes informal care a key behavioral driver of service use among older adults [31,32]. Finally, both international and Greek evidence suggest that preferences for private providers are often driven by individual preferences, previous experiences with the health system, or perceptions that private services offer higher-quality care, shorter waiting times, better communication with health professionals, and more personalized attention [33,34,35].

According to the conceptual approach developed by the World Health Organization (WHO) [20], health financing policy can be analyzed through three pillars: “*proposed objectives for health financing policy*”, “*framework for descriptive analysis of health financing systems and reforms*”, and “*fiscal constraints and other contextual factors*”. Here, we use this framework as a reference—an orientation tool rather than a prescriptive template—to situate Greece vis-à-vis objectives, functions, policies, and fiscal constraints in health financing. The three pillars do not operate independently; they interact and provide a practical way to structure the discussion, highlighting key policy choices, institutional arrangements, and implications, and helping identify long-standing issues and reform opportunities. Even where objectives are clearly articulated (pillar 1), results depend on how the financing functions—revenue collection, pooling, purchasing, and policy on benefit entitlements—are organized and governed (pillar 2), and on implementation capacity within fiscal and political constraints (pillar 3). Objectives guide—rather than determine—the organization of the financing functions (pillar 1 → pillar 2), while the organization and governance of those functions condition progress toward the objectives (pillar 2 → pillar 1). Fiscal and political constraints define the feasible space for both (pillar 3 → pillars 1–2). Finally, the health-financing policy objectives (pillar 1) serve as the criteria against which the performance of financing arrangements and the effects of reforms are assessed [20]. In Greece, this dynamic is visible in the gap between the stated aim of universal coverage and the frequent reliance on private providers for basic services.

## 3. Characteristics of the Greek Health System

The National Health System (ESY) was established in 1983 (Law 1397/1983) by the government at that time, fulfilling a campaign promise. It was a significant reform designed to create a public health system based on the Beveridge model, aiming to offer universal coverage for the population. The preamble to the founding law recognizes health as a social good, not a marketable product, with access provided on an equal basis regardless of economic or geographic location. It states that the exclusive responsibility of the state is to protect health through a unified, decentralized, and democratic service delivery mechanism. These principles express a clear social consensus that health care is a right, not a privilege, and that the state has a duty to ensure it is equally accessible. However, since its establishment, the system has not solely adopted the characteristics of the Beveridge model. Alongside state budget funding and full-time, exclusive employment of doctors in the public sector, social security mechanisms similar to the Bismarck model have been maintained, resulting in a hybrid system [36,37,38,39].

Although Greece aimed to combine the best features of different health models, it ended up with an institutional dualism that blurred the boundaries of political and social targeting. The parallel strengthening of the private sector and the erosion of the original ideological foundation have created a complex and often conflicting system, which exacerbates inequalities and shifts a substantial portion of the costs to citizens. Chronic shortages of personnel and infrastructure, long waiting times, and fragmented service delivery make reliance on the private sector almost unavoidable, shifting an increasing share of financing from pooled public funds to out-of-pocket payments and distancing the system from its original public mission [40]. In this context, the main concern is less about the label of Beveridge or Bismarck and more about the decline in financial protection, fair access, and the system’s capacity to provide effective care for acute episodes, chronic conditions, and the increasing needs of an aging population.

During the recent economic crisis within the framework of the Economic Adjustment Programme, the country was called to fulfill two main obligations in the health sector: reducing public spending to 6% of GDP and implementing an extensive reform program aimed at improving the efficiency of the system [41,42,43]. The interventions focused mainly on merging hospitals and creating EOPYY, which assumed financial responsibility and the role of sole public purchaser of health services, bringing together the powers and resources of the main insurance funds [44]. However, financial and administrative constraints limited its bargaining power, so that it did not function effectively as a monopsony, and its role in the stable and adequate financing of hospitals was undermined. At the same time, the lack of resources and the growing debts of hospitals, combined with the organization’s inability to meet its obligations, led to the gradual involvement of the Ministry of Health as a direct financier, alongside the institutionally prescribed role of EOPYY. The Ministry intervened with write-offs of claims and extraordinary measures to limit the consequences of the deficits [45]. At the same time, the state budget took on critical financing functions, reinforcing the overlap of roles and the diffusion of responsibilities.

Furthermore, in 2011, the KEN-DRG (Diagnosis-Related Groups) reimbursement system was introduced, replacing the retrospective per diem method used in hospitals. This prospective, case-based approach aimed to improve efficiency by aligning payments with standardized cases instead of per diem charges [41,46]. Greece adopted DRG relatively late compared to most EU countries. The initial objective was to compile a DRG list tailored to the Greek context by drawing on the experience of countries with similar service-delivery and financing arrangements [47]. However, unlike a fully developed DRG, KEN was based solely on the primary diagnosis, without taking into account the medical procedures and without using an automated classification tool and cost-adjustment process. Such weaknesses significantly limited its functionality, leading to distortions, a lack of transparency, and an inability to control actual costs [41,46,48]. Since then, the Greek DRG system has been revised twice (2019, 2021) in an effort to align it with international best practices, to establish a transparent and evidence-based reimbursement mechanism, to align funding with the actual cost of services, and to enhance the efficiency of the ESY [48].

These institutional shortcomings, together with role ambiguity and the lack of a clear, rules-based hospital-financing framework, are reflected in the current financing and access indicators analyzed in the following sections.

## 4. Health Expenditure Indicators

**Figure 1:**
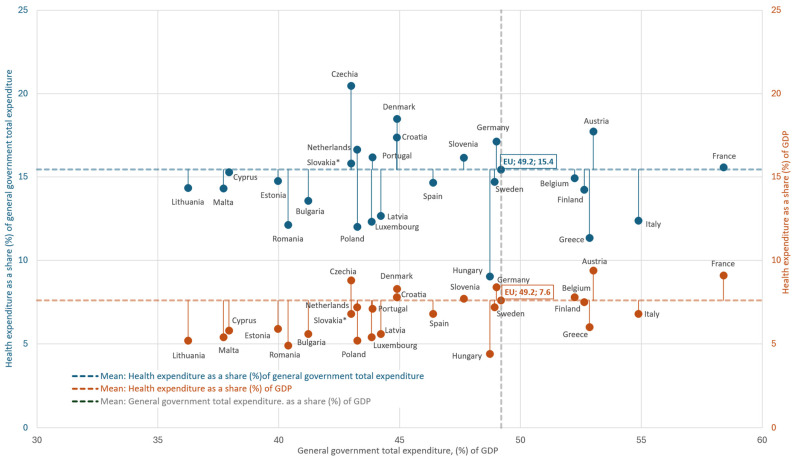
Health spending in the EU-27 (2022): relationship to government size. Points show each country’s general government expenditure (% of GDP, x-axis) with paired markers for health as a share of government spending (blue, left y-axis) and as a share of GDP (orange, right y-axis). Dashed lines denote EU-27 means; labels mark EU-27 values. Author’s design using Eurostat, 2022 [52] data; Ireland not included. * = provisional data. Greece allocates 11.3% of public expenditure to health (EU-27: 15.4%) and 6.0% of GDP to health (EU-27: 7.7%). Taken together, Greece combines a relatively large public sector with a comparatively small budget share for health.

The following data on key health financing indicators [20,49], position Greece significantly below the EU-27 average across critical parameters (data refer to the latest available year). In 2022, per capita health expenditure amounted to €1683, less than half the European average (€3684.55) [50]. In the same year, current health expenditure accounted for 8.50% of GDP, compared to 10.36% in the EU-27, with countries such as Germany (12.61%), France (11.88%), and Belgium (10.76%) investing significantly higher percentages. Public health expenditure in Greece amounted to 5.98% of GDP, significantly lower than the EU-27 average [51]. At the fiscal level, total public expenditure accounted for 52.9% of GDP (compared to 49.2% in the EU-27), but the share of health within public spending was only 11.3% (compared to 15.4% in the EU-27) (Figure 1) [52].

Given that public health spending reflects both available fiscal space and budgetary priorities, these figures may indicate that health has been accorded a relatively smaller share of public resources. Strengthening pooled funding could therefore require a budget reallocation toward health and—where feasible—higher public outlays [20,53]. Beyond the low level of public funding, the limited volume of in-kind health benefits indicates a historically weak redistributive capacity. In 2021, such benefits accounted for only 6% of adjusted disposable income, compared to 16.9% in the EU-27, placing Greece among the lowest-ranking member states [54]. This pattern partly reflects limited fiscal space for health and the disproportionate reliance on private payments—despite the existence of government and compulsory insurance schemes—both of which limit universality and financial protection.

**Figure 2:**
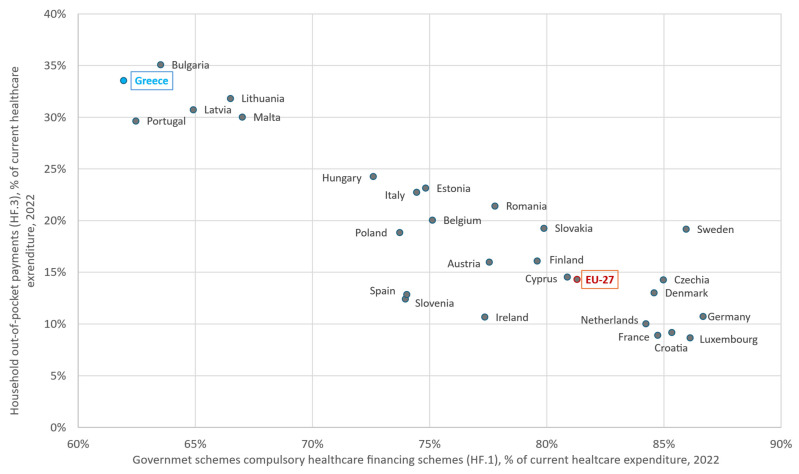
Government schemes and compulsory contributory health care financing schemes (HF.1) versus household out-of-pocket payments (HF.3) as a share of current health expenditure (2022), EU-27. Author’s design using sources: Refs. [50,55].

Table 2 shows that out-of-pocket household payments account for 33.5% of current health expenditure, confirming their predominant role in financing. It also reveals the fragmentation of health care financing schemes and collection mechanisms. In 2022 (the latest available year), only 62% of total health expenditure was publicly financed, the lowest share in the EU-27, versus an average of 81.3% (Figure 2). In Germany, the share reached 86.7%, with social insurance playing a dominant role [50,55]. In the same figure, countries with GDP levels closest to that of Greece (€207 billion in 2022), such as Portugal (€244 billion) and Hungary (€168 billion), keep out-of-pocket spending (30% and 24%, respectively) below the 33.5% observed in Greece, indicating that this high household burden cannot be explained by GDP alone.

The issue is not only caused by the economic conditions at the time but also reflects a long-standing inability to implement significant reforms to the financing model since ESY’s establishment in 1983 [58]. Despite significant interventions on the supply side, the financing pillar has remained virtually unchanged, operating on a “survival accounting” basis, often passing on costs to patients, either through their own means or through an informal and “shadow” private sector.

## 5. The Price of Inaction: High Private Spending, Low Protection

Availability of services alone does not ensure better efficiency, quality, or access on an equitable basis unless it is accompanied by a restructuring of financing aligned with the system’s objectives and changing social needs [59,60,61]. The health system must respond to demographic, epidemiological, geographical, and economic pressures in the current era [62]. Despite occasional upgrades, it continues to show efficiency deficits in both primary and secondary care [23,63,64,65]. In this context, the international literature finds no evidence that increased private participation improves efficiency; on the contrary, higher rates of public funding are associated with higher returns [7]. Although the Greek health system offers universal coverage, in practice equity in access remains elusive. Legal entitlements often do not translate into real access to services. As has been documented, even systems that claim universal coverage often restrict access in practice, mainly through limited benefit packages, uncontrolled private payments, weak redistribution across population groups or non-pricerationing measures such as waiting lists [18,20]. To address this gap, a “fourth” dimension—service access—can be added, capturing whether people can in practice obtain the services to which they are entitled [19].

**Figure 3:**
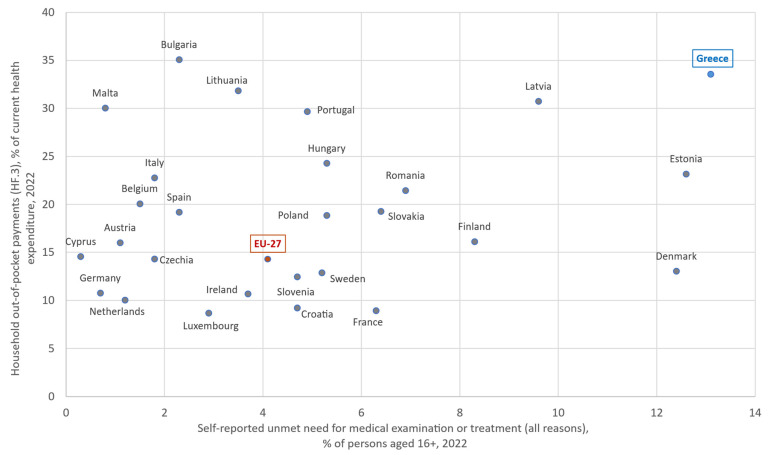
Self-reported unmet need for medical examination or treatment (all reasons, % of persons aged 16+, 2022) versus household out-of-pocket payments (HF.3) as a share of current health expenditure (2022), EU-27. Author’s design using sources: Refs. [57,66].

According to the WHO, financial protection is stronger when out-of-pocket (OOP) payments [56]—direct payments by users from the household’s primary income or savings at the time-of-service use or purchase, with no third-party payer involved—remain below 15–20% of total health expenditure [21]. In Greece, the share was 33.5% in 2022, more than twice the EU-27 average (15%) and comparable to countries such as Bulgaria, Latvia, and Lithuania. Public funding is among the lowest in Europe, while 9.8% of households incur catastrophic health expenditure exceeding 10% of their total budget or income [50], one of the highest rates in the EU-27. Out-of-pocket payments are mainly directed towards outpatient services, such as visits to private doctors, dental care and medicines, where public coverage is partial or non-existent. However, Greece stands out for its high OOP share in inpatient care (32% of OOP), versus about 9% on average in the EU-27 [50,57]. Figure 3 plots household out-of-pocket payments against self-reported unmet need for medical examination or treatment across the EU-27. Greece is located in the upper-right corner, representing an extreme point at the high end of the distribution.

### 5.1. Unmet Medical Care Needs and Inequalities

Greece has high rates of self-reported unmet needs for a medical examination or treatment (medical care), with 13.1% in 2023, relative to an average of 3.8% in the EU-27, ranking second, while in 2024 the rate moved into first place with 13.4% [67]. The gap between income groups is the highest in the EU-27 (18.7% in the lowest income quintile compared to just 2.9% in the highest), with the 15.8 percentage point difference highlighting the system’s long-standing failure to ensure access, with the greatest impact on the most vulnerable social groups [67]. That weakness is not limited to the financial dimension but is suggestive of systemic dysfunction. As has been pointed out, unmet needs mask the true level of catastrophic spending; if households had to cover them out of pocket, catastrophic spending would be even higher, particularly among poorer households [17]. Significant inequities are also observed between age groups, with the largest difference in the EU-27 recorded in 2023, with those over 65 reporting that they did not receive needed care at a rate 29.5 percentage points higher than those aged 16–44, revealing the system’s inadequate adaptation to the needs of the elderly [67]. At the same time, significant spatial disparities persist, especially in remote and underserved regions such as Eastern Macedonia and Thrace and the Aegean islands, where limited availability of basic services leads either to forgone care or to higher private spending [68]. Despite having the highest number of licensed physicians in the EU-27 (656 per 100,000 inhabitants, compared to the European average of 414) [69], their uneven geographical distribution and weak integration into the public system exacerbate inequities in access [70,71]. Finally, waiting lists, which are a key non-financial barrier to access [20], remain largely without systematic monitoring and evaluation—unlike in countries such as Denmark and Norway—causing delays in primary care, diagnostic tests, surgical procedures, and cancer care [72]. The absence of targeted measures to reduce them disproportionately affects low-income individuals, many of whom postpone or forgo treatment [73].

### 5.2. Dental Care: Limited Public Coverage

Public coverage of dental care is primarily limited to children up to the age of 18 and emergency care for adults, leaving regular care dependent on household financial capacity. However, the low recorded out-of-pocket expenditure on dental care may mask “silent” non-use of services rather than lower population need [74]. According to Eurostat’s 2023 data [67], Greece has the highest percentage of self-reported unmet dental care needs in the EU-27 (14.9% compared to the European average of 4.7%), with the majority attributed to financial reasons (86.7%). Social inequities are pronounced as 61.2% of people at risk of poverty reported unmet needs, compared to 22.6% of the rest of the population. The gap of 38.6 percentage points, the largest recorded in the EU-27, demonstrates persistent inequities in access to health services [67].

### 5.3. Informal Payments and Medicines: Burdens and Barriers

In Greece, patients’ out-of-pocket costs are a key barrier to access, even for prescription medicines that are partially covered by EOPYY [74], while non-prescription medicines add significant private costs. In 2022, only 51% of retail pharmaceutical expenditure was covered by public schemes, with the remaining 49% coming from direct payments -without any significant contribution from private insurance. In the EU-27, the corresponding public coverage averages about 70%, with significant variations between countries [50]. A low public share of retail pharmaceutical spending and high OOP may reflect resource allocation to compensate for structural deficiencies (e.g., maintaining extensive, under-resourced infrastructure), leaving fewer funds for medicines. Such patterns can be regressive, exposing poorer groups more to out-of-pocket costs [20].

Beyond the pharmaceutical sector, a particularly corrosive practice is the use of informal payments, commonly referred to as “envelope payments” or “under-the-table payments” (“fakelaki”), which are documented in public hospitals for services that are theoretically fully covered by the state. These unrecorded payments are sometimes a token of gratitude and sometimes an informal guarantee of better care [21,56,75]. They are not an isolated problem but a symptom of system-level structural failures. When public resources are wasted, informal payments proliferate and transparency erodes; tackling inefficiency is therefore integral to reducing inequalities in the use and provision of services and to improving financial protection, equity, and accountability [20,75,76].

According to research, in Greece, informal payments are reported in over 60% of health care encounters, a level also seen in several Central and Eastern European countries and in a number of former socialist countries in Europe [76,77]. Legalizing informal payments by transforming them into formal co-payments is not, by itself, a solution. Policy should prioritize the transparency of entitlements and obligations [20].

### 5.4. Challenges in Primary Health Care and the Hospital-Centered Model

Primary Health Care (PHC) constitutes the first point of entry into the health system and a central mechanism for patient navigation within it [78]. Despite a broad consensus on the pivotal role of primary care, Greece continues to devote a disproportionate share of expenditure to hospitals, which accounts for 45.3% of total health expenditure (EU-27: 36.4%). By contrast, ambulatory health care 15.8% (EU-27: 25%) and preventive care 0.7% (EU-27: 2.9%), remain underfunded and below the European averages [57]. Public underfunding of PHC leads to inadequate staffing, equipment shortages, and limited availability of services—especially outside normal working hours—resulting in increased out-of-pocket payments [79]. Despite declared intentions to strengthen it, PHC is often relegated to a complementary or secondary role, without strategic targeting, stable resources, or functional interconnection with other levels of care. As a result, it is unable to fulfill its core mission of prevention, timely intervention, and relieving pressure on hospitals. The system continues to operate without a unified referral mechanism, without universal implementation of the personal doctor institution and without a coordinated administrative structure, resulting in a lack of continuity of care and an accumulation of cases in hospitals [80]. By the end of June 2023, approximately 4.9 million people—about 55% of those eligible—had registered with a personal doctor [74]. Greece has the lowest, and only single-digit, percentage of general practitioners in the EU-27 (approximately 7% per 100,000 inhabitants) [69], while shortages in basic infrastructure and equipment and the uneven distribution of staff reinforce a vicious cycle of spatial inequities and social discontent [23,79]. Recent efforts to increase the number of general practitioners include a new standardized compensation system for primary care and educational measures specific to general practice, such as adding a family medicine module to the core curriculum, which will be implemented at over 75% of universities [74].

### 5.5. Deficits in Trust, Readiness, and Digital Maturity

According to the first international survey of patients in primary care (PaRIS) [81], Greece has one of the lowest levels of trust in the health system and in supporting institutions among participating countries. Among respondents with chronic conditions, fewer than half report confidence (versus an average of 62%), while 15% report a lack of confidence (compared to 9% on average). In general, users report only moderate satisfaction, reflecting accumulated fatigue of the system, gaps in meeting care needs, and low expectations [79,80,82]. At the same time, the findings on eHealth literacy and on individuals’ comfort in using eHealth information are a cause for concern. In Greece, just 9% of chronically ill individuals report comfort in using online sources for health information, compared with an average of 19% [81]. Despite progress in electronic prescribing and basic eHealth services, the health system’s digital maturity remains moderate. In 2024, Greece was ranked among the “Followers” with an overall score of 74% on the Composite eHealth Score, below the EU-27 average (78%). Low provider interoperability (only 27% send data electronically) and the limited use of digital services by citizens (approximately 75% have access to e-health records via eID, mainly for basic information) limit effective use of the infrastructure [83]. For example, only 3% of people with chronic conditions receive care in practices that can exchange electronic medical records, compared to an average of 57% in the OECD’s PaRIS [81]. Overall, these structural weaknesses limit the system’s ability to manage pressures, sustain effectiveness, and generate long-term gains.

## 6. Conclusions

Analysis of European data highlights the marked differences in the financing models of national health systems, particularly in terms of the balance between public and private resources. Greece operates a hybrid model in which private payments cover a disproportionate share of total expenditure, largely replacing public funding. The system remains trapped in a constant dilemma between public and private character, without having evolved into a comprehensive public scheme of universal coverage with a social orientation. Instead, it is silently sliding into a semi-private, two-tier model. The recent abolition of the full-time, exclusive employment status of doctors [74]—a key pillar of the public image of the National Health System—is shaping a new landscape, raising questions about the future direction of the system and the collective social agreement it represents. The financial crisis and the COVID-19 pandemic have starkly highlighted the weaknesses of the model, as spending cuts, understaffing, infrastructure deficiencies, and the increasing financial burden on citizens have exacerbated social inequities [64,84,85]. Although positive interventions, such as coverage for the uninsured, the establishment of the “family doctor” (later “personal doctor”) with the capitation payment system—which is based on the principle of equity and prioritizes the maintenance of overall health, rather than focusing solely on providing individual services for specific health problems [86]—and free preventive programs reinforce the system’s social mission [74,80,87], they remain incomplete and isolated, lacking an integrated financing framework.

The sustainability of the system requires stable and adequate public funding, efficient use of resources, and targeted investments in the health sector [5]. These are necessary conditions for universal and equitable access to quality preventive, therapeutic, and rehabilitative health services, without financial barriers and burdens at the point of use [88]. Conversely, resource waste and catastrophic health expenditures are symptoms of low-quality systems that exacerbate inequity and undermine sustainability [61]. When substantial public resources are wasted, progress on financial protection and transparency stalls, and the case for higher public spending loses credibility [20]. Greece has the highest level of private participation in health financing among the EU-27 countries, accompanied by particularly high rates of unmet needs and marked socioeconomic and spatial inequities in access. The persistent presence of informal payments, low trust in institutions, and low levels of satisfaction reveal systemic deficits in governance, transparency, and effectiveness, and highlight the gap between the goal of universal coverage and reality.

The failure to reform the financing model represents a missed opportunity to build a fair and effective public system. The critical question now is whether Greece will consciously choose to strengthen public funding or continue down the road toward one of the most privatized models in the EU, without a coherent strategy, letting funding shortfalls steadily erode efforts to improve provision. Given structural weaknesses—such as fragmented funding flows, unclear boundaries of responsibility among the actors involved, payment delays, and unequal resource distribution—it is useful to re-examine the system’s financing architecture, drawing on the WHO three-pillar framework as an orienting reference rather than a prescriptive template [20].

## 7. Recommendations

**Figure 4:**
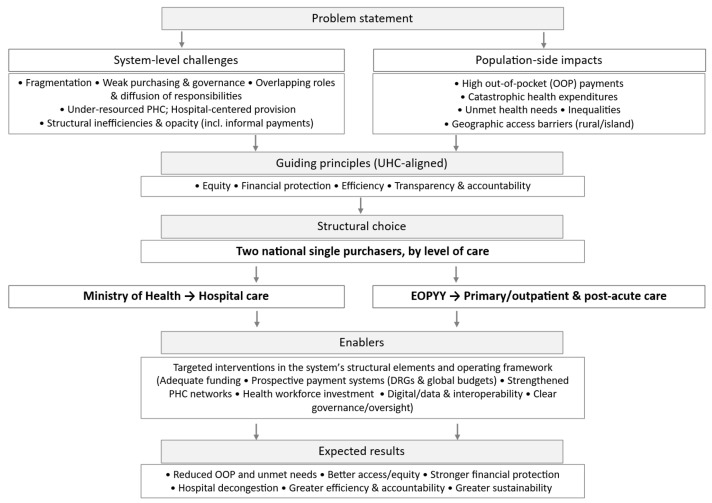
Conceptual flow diagram—from challenges and guiding principles to the structural choice, enabling policies, and expected results.

The central axis of the proposed reform is the establishment of two national single purchasers—one per level of care—of publicly covered services for a defined population/area [53], with a clear allocation of responsibilities and powers (Figure 4). The Ministry of Health is solely responsible for the financing and management of hospital care. It funds all publicly covered inpatient, surgical, and highly specialized services, and ensures a stable, planned, and transparent allocation of resources at the most costly and complex level of the system. In parallel, EOPYY focuses on financing primary health care (PHC) and post-hospital care, promoting prevention, universal access to core services, chronic disease management, and rehabilitation, while helping to ease pressure on hospitals.

Concentrating financial responsibility by level of care enables a unified resource-allocation strategy, greater efficiency and accountability, and access so that cost is not a barrier. Given that hospital care absorbs the largest share of health expenditure internationally, a clear and effective financing mechanism is needed, capable of controlling costs and rewarding continuous quality improvement [89]. In cases where public capacity—structural or operational—is insufficient, the private sector will be used in a targeted, complementary way through contracts with the Ministry in areas of persistent, well-documented need (e.g., ICUs, dialysis units, psychiatric care, cardiac surgery, maternity services).

In parallel, EOPYY is proposed to be transformed into a unified Primary Health Care (PHC) organization, acting as the sole funding body for all related services—from Health Centers and Local Health Units (TOMY) to pharmaceutical and outpatient care. The organization will collect resources from insurance contributions via the Hellenic National Social Security Entity (EFKA), while universal coverage for the uninsured will be ensured through the state budget. The new EOPYY will function as a public social health insurance organization with administrative and financial autonomy, enabling effective resource management and tailoring of services to regional needs (urban, rural, island, remote). The creation of a strong PHC network, funded mainly by progressive public revenues and offered free at the point of use, improving population health and reducing unnecessary hospitalizations [90]. Conversely, inadequate or distorted financing arrangements can undermine PHC effectiveness and push people toward more costly levels of care [86].

The success of the proposed reform requires targeted interventions in the structural elements of the system and its operating framework—particularly in human resources, data and digital health, medical technologies, and governance [8], to support daily operations as well as long-term sustainability. A key priority is strengthening the public health system by recruiting medical, nursing, and technical staff to fill existing gaps and fully use inactive infrastructure. The establishment of a tiered salary scale, by specialty and role requirements, may help attract new professionals and reduce the “brain drain” abroad. At the same time, regular staff and exit surveys can provide valuable input for workforce-retention policies [91].

The reorganization of health care services is a critical lever for improving efficiency. Implementing guidelines, clinical protocols, and interdisciplinary care models, strengthening day care, expanding the use of modern surgical techniques, improving discharge management, and developing rehabilitation and post-hospital care structures can increase the availability of hospital beds [92,93,94]. For example, in Belgium, Cyprus, and Croatia, about one quarter of hospital expenditures is directed to day-care activities [50]. Reducing the average length of stay by even one day is estimated to free up a significant number of beds, improve patient flow, and reduce hospital costs without compromising quality of care [92,93,94,95,96].

To fully realize this potential, a coherent institutional and financial framework is needed, one that sets not only the level and sustainability of spending but also patterns of service use through incentives embedded in reimbursement mechanisms [97]. According to the OECD [98], restructuring funding flows and adopting payment mechanisms that incentivize integrated care and strengthen coordination between levels of care can reduce fragmentation, a key driver of unnecessary hospitalizations and higher costs. At the hospital level, using prospective payment systems such as DRGs, combined with global budgets—an approach long recognized as necessary in Greece [46,47]—can align payments with actual costs and enhance efficiency, transparency, and strategic planning. European experience suggests this combination is among the most effective for containing costs and is applied in most EU countries, with variation in the intensity of implementation [99,100,101,102]. By contrast, retrospective payment systems, such as per diems and fee-for-service models, can fuel induced demand, leading to the excessive use of services and creating a greater financial burden on the system without corresponding health improvements [102].

Equally important is strengthening the linkage between primary and hospital care, so that they function as complementary parts of a unified health system. The effectiveness of this linkage requires primary care providers with clearly defined roles and responsibilities. Universal registration of the population with a first-contact physician can strengthen the management of health needs, reduce the unnecessary use of specialized services, and improve the allocation of available resources [103]. Although the institution of a “gatekeeping” physician has been repeatedly provided for in law in Greece, its implementation has remained incomplete, partly due to administrative weaknesses and fragmented support for the PHC system [79]. Experience from countries such as Norway, where universal registration with a general practitioner has been in place since 2001 and now covers more than 99% of people, suggests that a well-functioning referral system is associated with better health indicators and longer life expectancy [104]. In addition, building multidisciplinary teams and adopting common referral rules can help patients navigate the system, set clear criteria for moving between levels of care, and prevent delays or avoidable hospitalizations. Giving health professionals universal access to a single, integrated Electronic Health Record (EHR) can raise decision quality and make care more effective. At the same time, targeted investment in innovation, modern medical technology, and the digital transformation of the system, including the expansion of the National Telemedicine Network (EDIT) [74], can go a long way toward narrowing access gaps, especially on islands and in remote areas [105]. Finally, the introduction of professional management and merit-based administrative procedures, together with transparency and accountability tools—for example, centralized procurement control, digital tracking of funding flows, and public reporting of cost and quality indicators— is essential for the reform’s credibility and sustainability. Looking ahead, strengthening independent analytical capacity—whether within existing public bodies or through a dedicated institute in collaboration with university departments—to gather and analyze epidemiological data, conduct economic evaluations of new interventions and systematically monitor health budgets would further support these changes and provide a solid evidence base for health-financing decisions.

The aim is not to adopt a ready-made model but to implement an adaptable reform framework, oriented to explicit policy objectives and grounded in the existing organization and institutional arrangements of the health system [53]. That means reliable public funding, straightforward and transparent rules on entitlements and payments, and purchasers that coordinate rather than overlap. This viewpoint discusses a financing reform that could promote more favorable conditions for people-centered and outcome-focused care, especially for chronic conditions and an aging population, by stabilizing public funding and enabling more strategic purchasing within clear budgets constraints.

Health financing turns not only on resources but on political choices. In line with the WHO [21], it should ensure access to necessary, high-quality services while protecting users from financial hardship. In this sense, the proposed two-purchaser model is aligned with WHO universal health coverage goals, particularly those related to reducing unmet needs and strengthening financial protection. Through consistency, strategic vision, and social sensitivity, it is possible to ensure equitable access and meaningful coverage, protect citizens, and build trust in the health system.

## Figures and Tables

**Table 1:** The UHC cube: breadth (X), scope (Y), and depth (Z).

Axis	Description
X—Breadth	“The extent of the population that is entitled to services paid from pooled funds”
Y—Scope	“Inclusion or exclusion of specific services from coverage”
Z—Depth	“The cost that patients must incur to obtain these services”

Source: Implementing health financing reform: Lessons from countries in transition [20] (adapted for clarity/format).

**Table 2:** Greece: distribution of current health expenditure by main health financing schemes, 2022.

Source of Health Care Financing		Mode of Participation	Basic Method for Fund-Raising	Pooling	Share of Current Health Expenditure Greece
HF.1 Government schemes and compulsory contributory health care financing schemes	HF.1.1 Government schemes	Automatic: for all citizens/residents; or a specific group of the population (e.g., the poor) defined by law/government regulation.	Compulsory: budget revenues (primarily taxes).	National, sub-national, or program level.	30.2%
	HF.1.2 Compulsory contributory health insurance schemes	Mandatory: for all citizens/residents, or a specific group of the population defined by law/government regulation. In some cases, however, the enrolment requires actions to be taken by the eligible persons.	Compulsory: non-risk-related health insurance contribution. Insurance contributions may be paid by the government (from the state budget) on behalf of some non-contributing groups of the population, and the government may also provide general subsidies to the scheme.	National, sub-national, or by scheme; with multiple funds, extent of pooling will depend on risk-equalization mechanisms across schemes. Also depends on the extent of regulation of premium, and standardization of benefits across schemes.	31.7%
HF.2 Voluntary health care payment schemes		Voluntary.	Usually, non-income-related premiums (often directly or indirectly risk-related). Government may directly or indirectly (e.g., tax credits) subsidize.	Scheme level	4.3%
HF.3 Household out-of-pocket payment		Voluntary: willingness to pay of the household.	Voluntary: household disposable income and savings.	No inter-personal pooling	33.5%
HF.4 RoW financing schemes (non-resident)		Compulsory or voluntary.	Grants and other voluntary transfers by foreign entities.	Varies across programs.	0.2%

Note: Adapted from OECD/Eurostat/WHO, A System of Health Accounts 2011: Revised Edition, Table 7.2 [56]; non-applicable subcategories omitted; last column added (Greece, CHE shares, 2022). Percentages refer to Greece (2022) and are drawn from OECD Health Statistics; figures rounded to one decimal [50,57]. HF = Financing Schemes.

## Data Availability

All data used are publicly available from WHO, OECD and Eurostat as cited in the manuscript.
